# Rabin8 phosphorylated by NDR2, the canine early retinal degeneration gene product, directs rhodopsin Golgi-to-cilia trafficking

**DOI:** 10.1242/jcs.263401

**Published:** 2025-01-23

**Authors:** Theresa Fresquez, Beatrice M. Tam, Shannon C. Eshelman, Orson L. Moritz, Michael A. Robichaux, Dusanka Deretic

**Affiliations:** ^1^ Department of Ophthalmology and Visual Sciences, University of New Mexico, Albuquerque, New Mexico 87131, USA; ^2^ Department of Ophthalmology and Visual Sciences, University of British Columbia, Vancouver, BC V5Z 3N9, Canada; ^3^ Department of Ophthalmology and Visual Sciences, West Virginia University, Morgantown, West Virginia 26506, USA; ^4^ Department of Biochemistry and Molecular Medicine, West Virginia University, Morgantown, West Virginia 26506, USA; ^5^ Department of Cell Biology and Physiology, University of New Mexico, Albuquerque, New Mexico 87131, USA

**Keywords:** Cilium, Rab GTPases, Sensory Receptors, Rhodopsin

## Abstract

The Rab11–Rabin8–Rab8 ciliogenesis complex regulates the expansion of cilia-derived light-sensing organelles, the rod outer segments, via post-Golgi rhodopsin transport carriers (RTCs). Rabin8 (also known as RAB3IP), an effector of Rab11 proteins and a nucleotide exchange factor (GEF) for Rab8 proteins, is phosphorylated at S272 by NDR2 kinase (also known as STK38L), the canine early retinal degeneration (*erd*) gene product linked to the human ciliopathy Leber congenital amaurosis (LCA). Here, we define the step at which NDR2 phosphorylates Rabin8 and regulates Rab11-to-Rab8 succession in *Xenopus laevis* transgenic rod photoreceptors expressing human GFP–Rabin8 and its mutants. GFP–Rabin8 accumulated with endogenous Rabin8 at the Golgi-apposed exit sites (GESs), also known as the *trans*-Golgi network (TGN). Rabin8 mutants deficient in Rab11 binding prevented membrane association of GFP–Rabin8. GFP–Rabin8 and NDR2 kinase both interacted with the RTC-associated R-SNARE VAMP7 at the *trans*-Golgi and the GESs. Here, GFP–Rabin8 and the phosphomimetic GFP–Rabin8-S272E integrated into RTCs, which were subsequently functionalized by Rabin8 Rab8 GEF activity. Non-phosphorylatable GFP–Rabin8-S272A caused significant GES enlargement and deformation, possibly leading to unconventional membrane advancement toward the cilium, bypassing RTCs. Rabin8 phosphorylation loss due to an NDR2 gene disruption thereby likely causes dysfunctional rhodopsin Golgi-to-cilia trafficking underlying retinal degeneration and early-onset blindness.

## Teaser

**Summary:** Rabin8 phosphorylation by NDR2 (also known as STK38L) kinase, which is implicated in an inherited early retinal degenerative disease, affects rhodopsin Golgi-to-cilia trafficking.

## INTRODUCTION

Retinal rod photoreceptors are specialized neurons possessing a significantly enlarged primary cilium, the rod outer segment (ROS), filled with densely stacked membranous discs housing the light-sensing G-protein-coupled receptor (GPCR) rhodopsin and the associated phototransduction machinery. Incorporating new discs into the ROS is essential for maintaining photoreceptor health and function. Continuous ciliary expansion underlying ROS membrane renewal begins in the rod inner segment (RIS). The emergence of newly synthesized rhodopsin from the Golgi complex in the RIS leads to the assembly of a functional cilia-targeting complex (Kandachar et al., 2018; Wang et al., 2017). Golgi-to-cilia trafficking of rhodopsin crucially depends on the ability of the cilia-targeting complex to recognize the VxPx motif at the rhodopsin cytoplasmic tail (Concepcion et al., 2002; Deretic et al., 1998; Hollingsworth and Gross, 2013; Lodowski et al., 2013; Mazelova et al., 2009a; Takita et al., 2024; Tam et al., 2000). The ciliary-targeting signal (CTS) VxPx is a hotspot for mutations that interfere with rhodopsin transport, causing severe forms of autosomal dominant retinitis pigmentosa (adRP) and blindness (Aleman et al., 2008; Berson et al., 2002; Bessant et al., 1999; Sullivan et al., 2006). Regardless of the uniqueness of rhodopsin as the rod photoreceptor-specific GPCR, its ciliary trafficking is directed by the evolutionarily highly conserved interaction network of small GTPases, the Rab11–Rabin8–Rab8 ciliogenesis cascade, which parallels the Ypt32p–Sec2p–Sec4p yeast budding pathway (Bryant et al., 2010; Feng et al., 2012; Knodler et al., 2010; Mizuno-Yamasaki et al., 2012; Wang and Deretic, 2014; Westlake et al., 2011). Within this ancient exocytic module, a multifunctional protein Rabin8 (also known as RAB3IP) (Hattula et al., 2002), acts as a Rab11 (herein referring collectively to Rab11a and Rab11b protein forms unless indicated) effector and a guanine nucleotide exchange factor (GEF) for Rab8 (herein referring collectively to Rab8a and Rab8b protein forms unless indicated), thus mediating Rab conversion from Rab11, the penultimate Rab GTPase, to Rab8, the final Rab GTPase in the ciliary pathway (Feng et al., 2012; Knodler et al., 2010; Rink et al., 2005). Rabin8 is essential for ciliogenesis and mutations affecting its phosphorylation are implicated in an inherited retinal degenerative disease (Chiba et al., 2013; Goldstein et al., 2010).

Following the detection of the CTS VxPx in rhodopsin by the small GTPase Arf4 early in the ciliary pathway, large scaffold proteins with arrays of functional domains perform regulatory and effector functions that allow the ordered recruitment and activation of Arf and Rab GTPases in ciliary membrane progression. One such protein, the Arf GTPase-activating protein (GAP) ASAP1, mediates GTP hydrolysis on Arf4 and functions as an Arf4 effector that regulates the budding of post-Golgi rhodopsin transport carriers (RTCs), alongside Rab11a (Mazelova et al., 2009a; Reish et al., 2014; Wang et al., 2012), and the Rab11a effector FIP3 (also known as Rab11FIP3) (Wang and Deretic, 2015). At the later stage of ciliary trafficking, ASAP1, Rab11a and FIP3 recruit the next multifunctional regulator, the Rab8 GEF Rabin8, to the RTC budding sites (Wang and Deretic, 2015). The Rab11–FIP3 complex initiates Rabin8 preciliary trafficking and ciliogenesis (Walia et al., 2019). Notably, Rab11 engages several FIP family effectors localized in different trafficking pathways functioning in divergent cellular processes (Meyers and Prekeris, 2002). In ciliary receptor trafficking, Rabin8 binds to the non-canonical effector-binding site within the Rab11–FIP3 complex with a four-fold higher affinity over Rab11 alone, forming a unique Rab11a–FIP3–Rabin8 dual effector complex (Vetter et al., 2015a,b; Wang and Deretic, 2015). This Rab11a dual effector output might link the ciliary cargo, like rhodopsin, presented in the FIP3-context to Rabin8, which activates Rab8 for RTC fusion with the periciliary plasma membrane (Deretic et al., 1995; Mazelova et al., 2009a; Moritz et al., 2001; Wang et al., 2012). Rab8 is a known regulator of post-Golgi carrier fusion in other ciliated cells (Knodler et al., 2010; Nachury et al., 2007; Westlake et al., 2011).

Rabin8 functions in neurite outgrowth in Rab8 GEF activity-dependent and activity-independent manners, which are equally Rab11 dependent. Rabin8 contains several functional domains: an N-terminal TRAPPII trafficking complex-binding domain, a central Rab8 GEF domain, and the C-terminal Rab11-effector domain, which is connected to the GEF domain via a flexible linker (Vetter et al., 2015a). Rabin8 is phosphorylated at S272 within the linker domain by the NDR2 kinase (also known as STK38L) (Chiba et al., 2013). This site is well conserved in Rabin8 proteins, but not in the Sec2p yeast homolog that binds phosphatidylinositol-4-phosphate (PI4P) (Mizuno-Yamasaki et al., 2010), suggesting an evolutionary conserved Rabin8 regulation mechanism in higher eukaryotes. The crucial phosphorylation step allows intracellular progression of Rabin8 by advancing interactions with the successive components of the ciliary pathway. Rabin8 interacts with phosphatidylserine (PS), the most abundant negatively charged phospholipid in the cell, which is particularly enriched in the Golgi. The Rabin8 PS-binding domain in the linker region with a basic stretch rich in lysine residues (260-KTPFKKGHTRNKS-272) could serve as the PS-recognition motif (Caberoy et al., 2009). Thus, NDR2 phosphorylation of Rabin8 might directly regulate its PS-binding through the introduction of negative charges into the basic stretch, consequently switching its binding specificity from PS to the Sec15 component of the ciliary membrane trafficking Sec6/8 complex (also known as the exocyst) (Chiba et al., 2013; TerBush et al., 1996). The Sec6/8 complex is a common effector for Rab8 and Rab11, and is also found in the photoreceptors (Mazelova et al., 2009b). Rabin8 additionally interacts with the select ciliary modules including the TRAPPII trafficking complex, which possesses a centrosome-targeting ASH domain, and the BBSome complex, which consists of seven BBS proteins, mutations in which result in photoreceptor degeneration associated with the Bardet–Biedel syndrome (Cuenca et al., 2019; Nachury et al., 2007; Schou et al., 2014; Westlake et al., 2011; Ye et al., 2018). These interactions suggest a central role of Rabin8 in ciliary pathways of sensory receptors.

In terminally differentiated neurons, NDR1 (STK38) and NDR2 kinases regulate polarity, dendritic spine development and membrane trafficking through phosphorylation of Rabin8, the Rab11a effector FIP5 (also known as Rab11FIP5), as well as phosphatidylinositol 4 kinase β (PI4Kβ), which generates phosphatidylinositol 4-phosphate (PI4P) an essential phosphoinositide for Golgi complex formation and function (Rehberg et al., 2014; Rosianu et al., 2023; Ultanir et al., 2012; Yang et al., 2014). Notably, FIP5 is a canonical Rab11 effector, whereas Rabin8 and PI4Kβ bind the same non-canonical effector-binding site unique to Rab11 in a mutually exclusive manner (Burke et al., 2014; de Graaf et al., 2004; Meyers and Prekeris, 2002; Vetter et al., 2015b), indicating that neuronal NDR1 and NDR2 kinases selectively control endomembrane trafficking through the Rab11a dual effector output.

NDR2 kinase is the product of the canine early retinal degeneration (*erd*) gene (Berta et al., 2011). The SINE insertion in this gene affects multiple NDR2 functions, including the kinase activity (Goldstein et al., 2010). *Erd* is linked to the human inherited retinal degenerative disease Leber congenital amaurosis (LCA), characterized by early-onset blindness. NDR2 kinase regulates retinal interneuron proliferation and homeostasis and its absence in the *Ndr2* mouse knockout results in rhodopsin mislocalization (Leger et al., 2018), which is known to cause blindness in autosomal dominant retinitis pigmentosa. Although dysfunction of NDR2 kinase disrupts the rod ciliary pathway and causes early retinal degeneration, the impact of Rabin8 phosphorylation by NDR2 on ciliary trafficking in rod photoreceptors remains unknown. We hypothesized that phosphorylation of Rabin8 by NDR2 kinase is essential for ciliary trafficking of rhodopsin and ROS renewal. We found that the transgenic expression of non-phosphorylatable Rabin8 mutants led to significant disruption of the Golgi exit sites where NDR2 was localized, causing accumulation of endogenous Rabin8 and rhodopsin transiting to the ciliary base.

## RESULTS

### Human GFP–Rabin8 expressed in *X. laevis* transgenic rods accumulates with endogenous Rabin8 at the Golgi exit sites

**Fig. 1.**
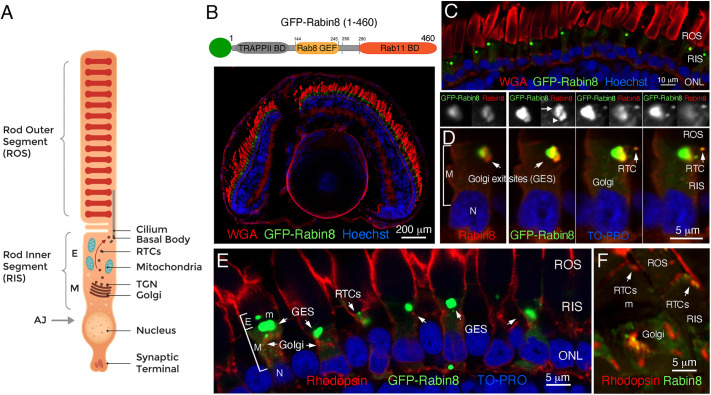
**Human GFP–Rabin8 expressed in *X. laevis* transgenic rods accumulates with endogenous Rabin8 at the GESs.** (A) Schematic of the rod photoreceptor cell. Rhodopsin transport carriers (RTCs) are formed at the Golgi exit sites (GESs, also known as the TGN) in the myoid region (denoted M) of the rod inner segment (RIS) and progress through the mitochondria-packed ellipsoid region (denoted E) to the base of the cilium, where they fuse with the RIS plasma membrane. (B) A schematic of the GFP–Rabin8 construct and a confocal optical section of transgenic *Xenopus* laevis eye expressing full-length human Rabin8–GFP fusion protein (green). Membranes are stained with wheat germ agglutinin (WGA, red). Nuclei are stained with Hoechst 33342 (blue). (C) Higher magnification image of the photoreceptor cell layer showing GFP–Rabin8-containing punctate structures in the RIS. (D) In transgenic photoreceptors GFP–Rabin8 (green) and endogenous Rabin8 (red) partially colocalize (yellow) on RTCs and at the GESs (arrows). The views above show separate red and green channels highlighting the area of GFP–Rabin8 colocalization with Rabin8 (arrow) and the neighboring area where only endogenous Rabin8 has accumulated (arrowhead). Nuclei (N) are stained with TO-PRO (blue). (E) GFP–Rabin8 localizes with rhodopsin, labeled with mAb 11D5 (red), in the Golgi in the myoid region (M) and on RTCs in the ellipsoid region (E). The dark edge of the clustered mitochondria (m) defines the border between the myoid and ellipsoid. Note that mAb 11D5 inconsistently stains ROS membranes, as previously reported. ONL, outer nuclear layer containing photoreceptor nuclei. (F) Non-transgenic frog retina labeled with anti-rhodopsin 11D5 (m) (red), and anti-Rabin8(r) (green). Images shown representative of two separate transgenesis experiments (∼30 tadpoles).

As indicated in Fig. 1A, during polarized cilia-directed trafficking in the photoreceptor RIS, rhodopsin exits the photoreceptor Golgi or trans-Golgi network (TGN) in the myoid region (M) within RTCs, which then enter the ellipsoid region (E), traverse the mitochondria-rich area and fuse with the plasma membrane at the ciliary base. Rabin8 is fundamental to ciliogenesis. Therefore, we sought to elucidate the role of its phosphorylation by NDR2 kinase in rhodopsin ciliary targeting and RTC budding in rods. Because *Xenopus* and human Rabin8 are 78% identical, and the consensus motif of NDR substrates (HxRxxS/T) is highly conserved (Vetter et al., 2015b), we expressed human Rabin8 as a GFP fusion protein in transgenic *X. laevis* rod photoreceptors (Fig. 1B), as previously described (Kandachar et al., 2018; Mazelova et al., 2009a; Tam et al., 2013, 2006). Confocal microscopy revealed that GFP–Rabin8 predominantly localized and accumulated in distinctive large punctate structures in the RIS (Fig. 1C), possibly due to its high expression level relative to its Rabin8 endogenous partners. The Golgi-apposed structures localized in the myoid, facing the cilia are termed Golgi exit sites (GESs), based on the directionality of the Golgi-to-cilia rhodopsin trafficking in photoreceptors, as depicted in Fig. 1A. The GESs containing GFP–Rabin8 likely correspond to the enlarged TGN, however, the species-specific reactivity of available antibodies to the TGN markers precludes its exact validation in the frog retina. GFP–Rabin8 accumulated at the GESs (Fig. 1D, green) and was partially colocalized with endogenous Rabin8 detected by the anti-Rabin8 antibody, which accumulated nearby (Fig. 1D, red and upper panels), probably due to the limited displacement of endogenous Rabin8 by overabundant GFP–Rabin8. The build-up of endogenous Rabin8 and GFP–Rabin8, both substrates of NDR2 kinase, points to the likely localization of their shared endogenous partner NDR2 at the Golgi or GESs. Importantly, GFP–Rabin8 localized with rhodopsin in the myoid region in the Golgi, and in the ellipsoid region on RTCs (Fig. 1E). Endogenous Rabin8 localizes in distinct puncta along the rhodopsin-laden Golgi in the non-transgenic retina (Fig. 1F), as previously reported (Kandachar et al., 2018).

### Rab11 regulates intracellular membrane localization of Rabin8, whereas its Rab8 GEF activity shapes functional RTCs

**Fig. 2.**
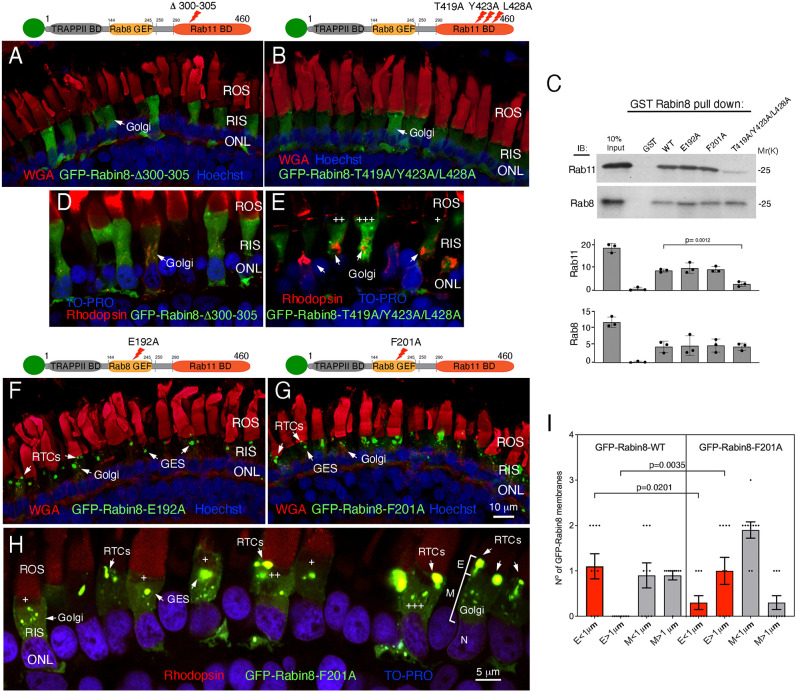
**Differential expression of human GFP–Rabin8 mutants deficient in Rab11 interactions or Rab8 GEF activity.** (A,B) Confocal optical sections of transgenic retinas expressing GFP–Rabin8 mutants Δ300–305 or T419A/Y423A/L428A (green), affecting Rabin8–Rab11 interactions. Membranes are stained with WGA (red). Nuclei are stained with Hoechst 33342 (blue). (C) GST–Rabin8-WT and its mutants E192A, F201A and T419A/Y423A/L428A, or GST alone, were incubated with purified Rab11, in the presence of GTPγS, or purified Rab8, in the presence of GDPβS. Bound Rab11 and Rab8 were detected by immunoblotting (IB), quantified using Image Lab 6.1, normalized for equal loading and. Rabin8-T419A/Y423A/L428A mutant binding to Rab11 was significantly different from the Rabin8-WT (*P*=0.0012, *n*=3). Data are presented as the means±s.e.m. *P*-values calculated using a two-tailed unpaired *t*-test. (D,E) Confocal optical sections of transgenic retinas expressing GFP–Rabin8 mutant Δ300–305 (D, green), or T419A/Y423A/L428A (E, green) labeled with anti-rhodopsin mAb 11D5 (red). Nuclei are stained with TO-PRO (blue). Arrows in E indicate rhodopsin-laden Golgi devoid of Rabin8 mutant deficient in Rab11 binding. Different transgene expression levels are indicated by+(low), ++ (medium) and +++ (high). (F,G) Confocal optical section of transgenic retinas expressing GFP–Rabin8 mutants E192A or F201A affecting their Rab8 GEF activity. Membranes and nuclei are stained as in A,B. (H) Confocal optical section of a transgenic retina expressing the GFP–Rabin8-F201A mutant (green) labeled with anti-rhodopsin mAb 11D5 (red). Nuclei are stained with TO-PRO (blue). Arrows indicate considerably enlarged RTCs accumulating in the ellipsoid region (E). (I) After calculating S_v_^GFP-Rabin8^ using the number of intersections of GFP-positive membranes with the line grid of a known size, the number and size of GFP-positive membranes intersecting the grid in the ellipsoid (denoted E) and the myoid (M) of photoreceptors expressing GFP–Rabin8-WT (*n*=10, Fig. 1E) and GFP–Rabin8-F201A (*n*=10, panel H) were quantified. Data are presented as the means±s.e.m. Statistical analysis was performed as in C. Images shown representative of two separate transgenesis experiments (∼30 tadpoles for each transgene).

To determine the effect of Rabin8 mutations that disrupt interactions with Rab11 and Rab8 and its phosphorylation by NDR2 kinase on rhodopsin trafficking, we generated appropriate Rabin8 mutants affecting its specific interaction domains. To disrupt the binding of Rabin8 to Rab11, we first expressed the GFP–Rabin8-Δ300-305 mutant reported to have a dominant-negative effect on neurite outgrowth (Homma and Fukuda, 2016). Confocal microscopy revealed that GFP–Rabin8-Δ300-305 was entirely cytosolic (Fig. 2A); however, the six-amino-acid deletion could have also disrupted Rabin8 dimerization, which is essential for its function (Guo et al., 2013; Vetter et al., 2018). The triple mutant Rabin8-T419A/Y423A/L428A has been reported to disrupt Rab11 binding, but not Rabin8 dimerization (Vetter et al., 2015a). We thus introduced T419A, Y423A and L428A mutations into GFP–Rabin8. The GFP–Rabin8-T419A/Y423A/L428A mutant was also completely cytosolic (Fig. 2B), indicating that Rab11 is crucial for Rabin8 membrane association in the RIS. GST pulldowns confirmed that Rabin8-T419A/Y423A/L428A mutant binding to Rab11 was significantly diminished compared to that of wild-type Rabin8 (Rabin8-WT; *P*=0.0012, *n*=3) (Fig. 2C). Variable expression of the GST–Rabin8-Δ300-305 mutant, possibly due to misfolding, prevented reliable analysis of Rabin8-Δ300-305–Rab11 binding. Like Rabin8-WT, Rabin8-T419A/Y423A/L428A mutant interacted with Rab8 (Fig. 2C). Notably, the cytosolic GFP–Rabin8-Δ300-305 and GFP–Rabin8-T419A/Y423A/L428A triple mutant (Fig. 2D,E, green) did not colocalize with rhodopsin, which was predominantly concentrated in the Golgi (Fig. 2D,E, red). In higher expressing cells (Fig. 2E, +++), GFP–Rabin8-T419A/Y423A/L428A accumulated around the Golgi but did not appear membrane-bound. These experiments indicate that binding to Rab11 is essential for Rabin8 membrane association and correct intracellular localization.

Next, we expressed GFP–Rabin8-E192A and GFP–Rabin8-F201A mutants deficient in Rab8 GEF activity (Vetter et al., 2018). Both mutants localized at the GESs and on RTCs (Fig. 2F,G). GST pulldowns showed that the E192A and F201A mutations did not alter the ability of Rabin8 to interact directly with Rab8 and Rab11 (Fig. 2C). GFP–Rabin8-F201A colocalized with rhodopsin at the base of the cilium, on RTCs in the ellipsoid (E), and in the Golgi and the GESs in the myoid (M) (Fig. 2H). In cells expressing low and medium levels of GFP–Rabin8-F201A (Fig. 2H,+and ++) the mutant localized in the Golgi, GESs and RTCs. Cells expressing high levels of GFP–Rabin8-F201A mutant (Fig. 2H, +++) showed a buildup of the fusion protein in enlarged RTCs, possibly due to its high expression level relative to endogenous Rab8, which binds to but cannot be activated by the GFP–Rabin8-F201A mutant. Enlarged RTCs were heavily labeled with an anti-rhodopsin antibody, indicating the newly synthesized rhodopsin was accumulating there instead of being delivered to the ROS via Rab8-mediated fusion at the ciliary base. RTCs appeared to be substantially larger in the GFP–Rabin8-F201A-expressing cells (Fig. 2H) than in the cells expressing GFP–Rabin8-WT (Fig. 1E). To ascertain that the expression of the GFP–Rabin8-F201A mutant caused RTC enlargement, we measured and quantified GFP–Rabin8-containing membranes in a volume of the cell ellipsoid (where RTCs are localized) and myoid (where the Golgi and GESs are localized). We calculated the S_v_^GFP-Rabin8^, which represents a measure of the surface occupied by GFP–Rabin8-containing membranes in a volume of the cell cytoplasm (Deretic et al., 2004; Mazelova et al., 2009a). The S_v_^GFP-Rabin8^ was determined by assessing the number of intersections of GFP-positive membranes with the line grid of a known size (Weibel and Bolender, 1973) in ten photoreceptor cells from each phenotype. Owing to the nature of transgenic *Xenopus*, each photoreceptor can be considered a separate data point (i.e. biological replicate) because expression level can vary significantly from cell to cell. The S_v_^GFP-Rabin8-F201A^ was not significantly different from the S_v_^GFP-Rabin8-WT^ in the ellipsoid (0.21±0.04, versus 0.18±0.04, *n*=10, *P*=0.59), or in the myoid (0.35±0.03, versus 0.40±0.06, *n*=10, *P*=0.51). However, the size distribution of GFP–Rabin8-bearing membranes intersecting the grid used for S_v_^GFP-Rabin8^ calculations significantly differed in the Rabin8-WT and the F201A mutant. The number of Rabin8-positive membranes of less than 1 µm, likely representing RTCs, was significantly higher in the ellipsoid of the photoreceptors expressing GFP–Rabin8-WT than in the GFP–Rabin8-F201A mutant (1.10±0.26, versus 0.30±0.14, *P*=0.0201, *n*=10) (Fig. 2I). As RTCs are only localized in the ellipsoid and do not exceed 300 nm, no Rabin8-positive membranes larger than 1 µm intersected the grid in the ellipsoid of the photoreceptors expressing GFP–Rabin8-WT; however, a significant number of Rabin8-positive membranes larger than 1 µm intersected the grid in the ellipsoid of GFP–Rabin8-F201A mutant (0.0±0.0, versus 0.50±0.21, *P*=0.0035, *n*=10). In the myoid, GESs larger than 1 µm were generally more frequent in cells expressing GFP–Rabin8-WT than in GFP–Rabin8-F201A mutants where they were present only in cells expressing low transgene levels (Fig. 2H,I). Collectively, these data indicate that the sufficient expression of GFP–Rabin8-F201A mutant deficient in Rab8 GEF activity blocks rhodopsin trafficking and causes a build-up of significantly enlarged RTCs in the RIS ellipsoid. This phenotype closely resembles that seen with the Rab8-T22N GTP-binding-deficient mutant, which caused the accumulation of irregular RTCs at the ciliary base (Moritz et al., 2001).

### Rabin8 is phosphorylated by NDR2 kinase at the GESs, before activating Rab8 on RTCs

**Fig. 3.**
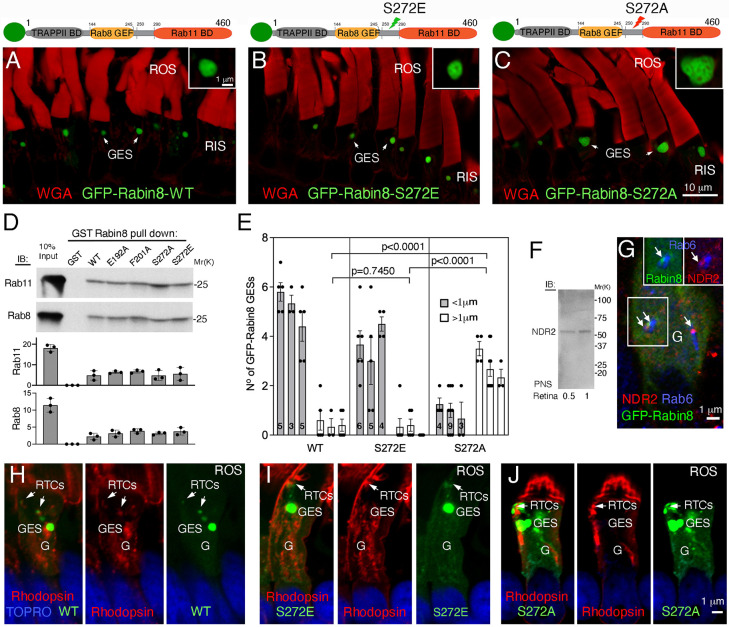
**Rabin8 phosphorylation likely occurs at the GESs.** (A–C) Confocal optical sections of transgenic retinas expressing GFP–Rabin8-WT (A, green), or the GFP–Rabin8-S272E (phosphomimetic) (B) or GFP–Rabin8-S272A (non-phosphorylatable) mutants (C). Insets in A–C show magnified GESs. (D) GST–Rabin8-WT and its mutants E192A, F201A, S272E, S272A or GST alone, were incubated with purified Rab11, in the presence of GTPγS, or purified Rab8 in the presence of GDPβS. Bound Rab11 and Rab8 were detected by immunoblotting (IB), and quantified as in Fig. 2C. There are no significant differences between GST-Rabin8 fusion proteins (two-tailed unpaired *t*-test). (E) The number and size of GESs were quantified in the retinas of transgenic animals expressing GFP-Rabin8-WT (*n*=3), GFP–Rabin8-S272E (*n*=3) or GFP–Rabin8-S272A (*n*=3). Data are presented as the means±s.e.m. The number of individual photoreceptor cells is indicated inside the bars. Statistical analysis was performed using a two-tailed unpaired *t*-test. (F) Immunoblot of the PNS prepared from frogs *Rana berlandieri* probed with anti-NDR2 antibody. Blot shown representative of three repeats. (G) A transgenic photoreceptor expressing GFP–Rabin8 (green) labeled with anti-NDR2 (red) and anti-Rab6 antibody (blue). Arrows point to the localization of GFP–Rabin8 and NDR2 at the tips of the *trans*-Golgi cisternae delineated by Rab6. Insets show separate red and green channels highlighting the area of GFP–Rabin8 colocalization with NDR2 (arrows). (H–J) Confocal optical section of transgenic retinas expressing GFP-Rabin8-WT, or S272E or S272A mutants (green) labeled with anti-rhodopsin mAb 11D5 (red). Nuclei are stained with TO-PRO (blue). Arrows in H and I indicate RTCs containing GFP–Rabin8-WT (H) or the GFP–Rabin8-S272E phosphomimetic (I). Arrows in J indicate RTCs surrounded by large structures containing non-phosphorylatable GFP–Rabin8-S272A, which also accumulated at the GESs. G labels, Golgi. Images shown representative of two separate transgenesis experiments (∼30 tadpoles for each transgene).

To investigate the role of NDR2 kinase phosphorylated Rabin8 in rhodopsin trafficking, we expressed phosphomimetic GFP–Rabin8-S272E and the non-phosphorylatable GFP–Rabin8-S272A human Rabin8 mutant fusion proteins in *X. laevis* rods. Confocal microscopy revealed similar GES sizes in the transgenic retinas expressing GFP–Rabin8-WT and the GFP–Rabin8-S272E phosphomimetic (Fig. 3A,B and insets). By contrast, the non-phosphorylatable GFP–Rabin8-S272A mutant accumulated in noticeably expanded GESs with tubulo-vesicular contents (Fig. 3C and inset). GST pulldowns showed that the Rabin8 S272E and S272A mutations did not significantly affect binding to either Rab8 or Rab11 (Fig. 3D). To ascertain that the enlarged GESs are not due to higher GFP–Rabin8-S272A expression levels compared to GFP–Rabin8-S272E mutants, we examined retinal confocal sections from GFP–Rabin8-S272A and GFP–Rabin8-S272E mutants, and compared them to GFP–Rabin8-WT. The sections from three transgenic animals from each group containing approximately ten photoreceptor cells were chosen to account for the natural mosaicism of the transgene expression. The mean number of Rabin8-positive enlarged GESs (>1 µm) in cells expressing the GFP–Rabin8-S272E phosphomimetic was low and not significantly different from that seen for GFP–Rabin8-WT (0.34±0.26, versus 0.43±0.26, *n*=3, *P*=0.7450) (Fig. 3E). In contrast, the mean number of Rabin8-positive enlarged GESs (>1 µm) in non-phosphorylatable GFP–Rabin8-S272A-expressing cells (2.87±0.39, *n*=3) was higher and significantly different from both that for the GFP–Rabin8-WT and the GFP–Rabin8-S272E mutant (*P*<0.0001) (Fig. 3E). Thus, although the GESs occasionally differ in size in transgenic retinas expressing GFP–Rabin8-S272E mutant and GFP–Rabin8-WT (see also Fig. 1E), the enlarged GESs do not appear to be a consequence of higher transgene overexpression, but rather a phenotype of GFP–Rabin8-S272A mutants.

To determine whether the photoreceptor NDR2 kinase localized in the Golgi area where it could phosphorylate Rabin8 and affect the GES size and shape, we employed an antibody against NDR1 and NDR2 (anti-NDR1/2 antibody) raised using a large fragment of human NDR2 protein, making it likely to recognize NDR2 in the frog retina as *Xenopus* and human NDR2 are 92% identical. Anti-NDR1/2 antibody recognized a single protein of the correct size (∼55 kDa) in the frog retinal post-nuclear supernatant (PNS) (Fig. 3F). Using this antibody, we found that both NDR2 kinase and GFP–Rabin8-WT localized at the tips of the trans-Golgi cisternae positive for Rab6 (herein referring collectively to all Rab6a–Rab6c forms) (Fig. 3G), where regulators of early stages of ciliary trafficking Arf GEF GBF1 and Arf4 also colocalize with rhodopsin (Wang et al., 2017). We thus concluded that the anti-NDR1/2 antibody detects the frog photoreceptor NDR2 because: (1) the antibody specifically recognized a single 55 kDa protein without cross-reacting with other retinal proteins, (2) immunolocalization of the protein appeared comparable to established components of the ciliary trafficking pathway, (3) the mouse *Ndr2* knockout causes rhodopsin mislocalization indicating that NDR2 is expressed in retinal photoreceptors within rhodopsin trafficking pathway, and (4) we observed NDR2 kinase within the inner nuclear layer (data not shown), where it is known to cause cell proliferation (Leger et al., 2018). Labeling with an anti-rhodopsin antibody established that GFP–Rabin8-WT and GFP–Rabin8-S272E colocalized with rhodopsin on RTCs (Fig. 3H,I). By contrast, in some GFP–Rabin8-S272A-expressing cells, large aberrant structures containing non-phosphorylatable Rabin8 surrounded but were excluded from rhodopsin-positive RTCs at the base of the cilium (Fig. 3J). The RTCs devoid of the GFP–Rabin8-S272A mutant substantially differ from the GFP–Rabin8-F201A phenotype, which causes accumulation of GFP–Rabin8–rhodopsin-positive membranes at the ciliary base.

**Fig. 4.**
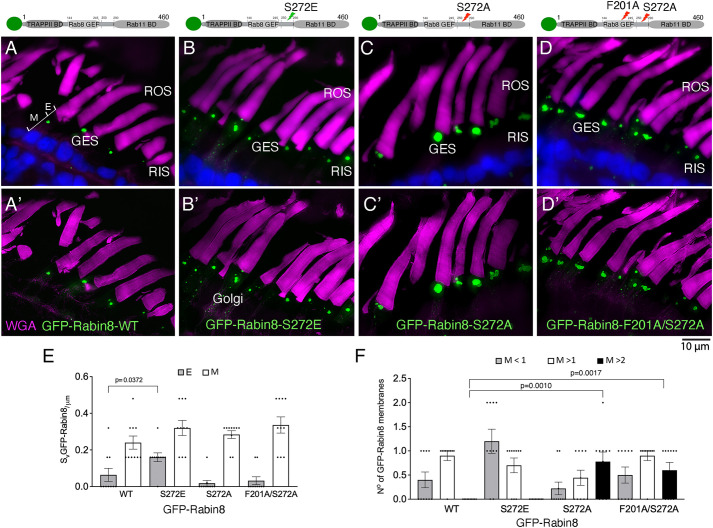
**Rabin8 phosphorylation at the GESs precedes Rab8 activation on RTCs.** (A–D) Reconstructed SIM images of transgenic retinas expressing GFP–Rabin8-WT (A, green), the GFP–Rabin8-S272E (phosphomimetic) (B), GFP-Rabin8-S272A (non-phosphorylatable) mutants (C) or the GFP–Rabin8-F201A/S272A double mutant (D) Membranes are stained with WGA (magenta). (A′–D′) Parallel SIM images followed by 3D deconvolution. Images shown representative of two separate transgenesis experiments. (E) S_v_^GFP-Rabin8^ was calculated by the number of intersections of GFP-positive membranes with the line grid in 10 photoreceptors from each phenotype. A two-tailed unpaired *t*-test shows that GFP–Rabin8-S272E value significantly differs from the GFP–Rabin8-WT value (*P*=0.0372, *n*=10) in the ellipsoid (denoted E). (F) The number and size of GFP-positive membranes intersecting the grid in the myoid (M) were quantified. Statistical analysis was performed as in E. Compared to GFP–Rabin8-WT, a significant number of Rabin8-positive membranes larger than 2 µm intersected the grid in the myoid of the photoreceptors expressing the non-phosphorylatable mutants GFP–Rabin8-S272A (*P*=0.0010, *n*=10) and F201A/S272A (*P*=0.0017, *n*=10) containing enlarged GESs. Data are presented as the means±s.e.m.

Analysis by super-resolution structured illumination microscopy (SR-SIM) further corroborated the differences between the two phosphorylation mutants; at comparable expression levels, the GFP–Rabin8-S272E phosphomimetic localized at the Golgi, GESs and on RTCs, similar to GFP–Rabin8-WT (Fig. 4A,B); however, GFP–Rabin8-S272A mutant protein predominantly accumulated at enlarged GESs (Fig. 4C). To further evaluate the non-phosphorylatable Rabin8 mutant, we considered the dramatic phenotype of the GFP–Rabin8-F201A mutant deficient in Rab8 GEF activity and hypothesized that the introduction of the S272A mutation would arrest the double mutant at the GESs and prevent the RTC enlargement phenotype caused by the F201 mutation. We thus generated a GFP–Rabin8-F201A/S272A double mutant and analyzed its intracellular localization in transgenic rods. Indeed, unlike GFP–Rabin8-F201A, the double mutant accumulated in the enlarged GESs closely resembling the dominant GFP–Rabin8-S272A phenotype (Fig. 4D). Corresponding 3D-deconvolution images (Fig. 4A′–D′), showed that, unlike the WT and S272E mutant, S272A and F201A/S272A non-phosphorylatable mutants had hyperextended GESs comprising extensive membrane networks. To differentiate the mutant phenotypes, we calculated the S_v_^GFP-Rabin8^ for all mutants (Fig. 4E). In the ellipsoid, S272A, and F201A/S272A non-phosphorylatable mutants did not significantly differ from the WT. By contrast, S_v_^GFP-Rabin8-S272E^ was significantly different from the S_v_^GFP-Rabin8-WT^ (0.16±0.02 versus 0.06±0.04, *P*=0.0372, *n*=10), indicating increased post-Golgi membrane budding in the Rabin8-S272E phosphomimetic. Although no prominently enlarged GFP–Rabin8-positive membranes (>2 µm) intersected the grid in the myoid of the photoreceptors expressing GFP–Rabin8-WT or GFP–Rabin8-S272E (Fig. 4F), a significant number of enlarged Rabin8-positive membranes (>2 µm) intersected the grid in the myoid of the photoreceptors expressing the non-phosphorylatable mutants GFP–Rabin8-S272A (0.0±0.0 versus 0.78±0.21, *P*=0.0010, *n*=10) and F201A/S272A (0.0±0.0 versus 0.60±0.15, *P*=0.0017, *n*=10). These data support the notion that enlarged GESs are a phenotype of both non-phosphorylatable GFP–Rabin8-S272A and GFP–Rabin8-F201A/S272A mutants. Moreover, they imply that the Rabin8 phosphomimetic successfully exits the GESs and reaches the ciliary base, whereas the non-phosphorylatable F201A/S272A double mutant is arrested at the GESs preventing the RTC enlargement phenotype caused by the unproductive Rabin8-F201A–Rab8 interaction, which blocks RTC fusion.

### Expression of non-phosphorylatable GFP–Rabin8-S272A mutant causes significant GES enlargement

**Fig. 5.**
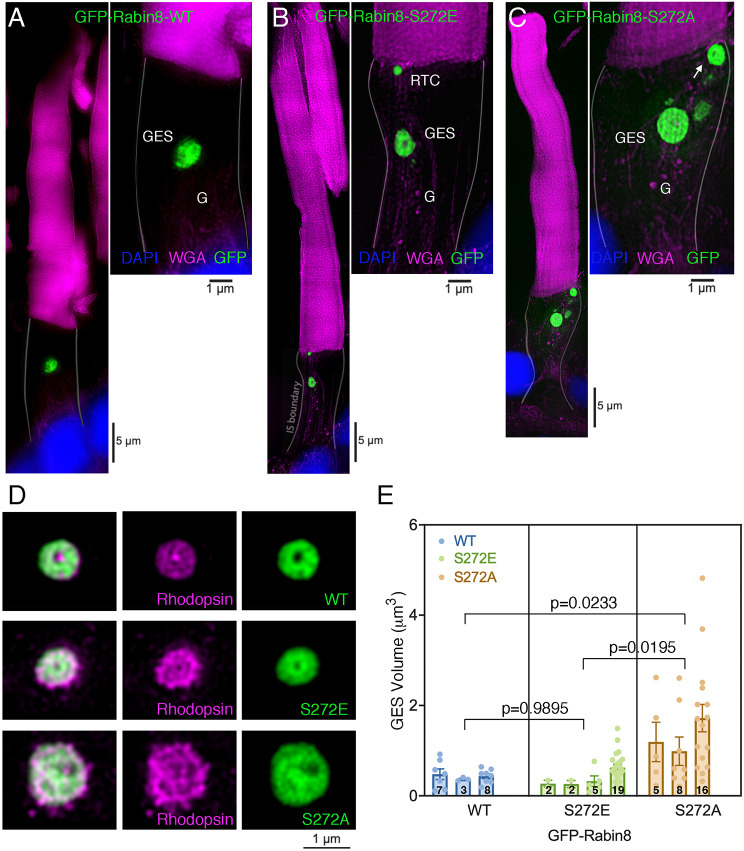
**The expression of the non-phosphorylatable GFP–Rabin8-S272A mutant causes significant GES enlargement and tubulation.** (A–C) Reconstructed SIM images of transgenic photoreceptor cells expressing GFP–Rabin8-WT (A, green), or GFP–Rabin8-S272E (phosphomimetic) (B) or GFP–Rabin8-S272A (non-phosphorylatable) mutant (C). Membranes are stained with WGA (magenta). Whereas the phosphomimetic mutant associates with RTCs and its GES resembles the WT (A,B), the non-phosphorylatable mutant is found in the expanded GES and the large membranous structures at the ciliary base (C, arrow), comparable to those Fig. 3J. G labels, Golgi. Images shown representative of two separate transgenesis experiments. (D) Selected magnified GES images of Rabin8-WT, and S272E and S272A mutants labeled with anti-rhodopsin antibody (magenta). The morphology of the WT GES and the S272E mutants is similar, whereas the GES size and the rhodopsin-bearing membrane content in S272A appear perturbed. (E) For each transgene, the GES volume was measured using Fiji/ImageJ in animals from WT (*n*=3), S272E (*n*=4) or S272A (*n*=3). The 3D GES volume in GFP–Rabin8-S272A-expressing cells significantly differed from that for the GFP–Rabin8-WT (*P*=0.0233) and the GFP–Rabin8-S272E mutant (*P*=0.0195). Data are presented as the means±s.e.m. *P*-values calculated using a nested one-way ANOVA with Tukey's post-hoc test. The number of individual photoreceptor cells is indicated inside the bars.

A more detailed SIM examination of individual rods showed that the GFP–Rabin8-WT and GFP–Rabin8-S272E phosphomimetic localized at similarly shaped GESs (Fig. 5A,B). As above, Rabin8-S272E localized on post-Golgi membranes consistent with RTCs, indicating its proper association with the ciliary pathway (Fig. 5B). In cells expressing GFP–Rabin8-S272A at similar levels, GESs appeared as large tubulo-vesicular membranous aggregates (Fig. 5C). Notably, some membrane structures that were an order of magnitude larger than RTCs and contained non-phosphorylatable GFP–Rabin8-S272A were located near the ciliary base (Fig. 5C, arrow). These resembled the rhodopsin-devoid structures surrounding RTCs seen in Fig. 3J, which could have reached the base of the cilium by an atypical trafficking pathway rather than by association with RTCs. By SIM analysis, rhodopsin was detected in the GESs of all Rabin8 transgenics (Fig. 5D). Upon close inspection, the doughnut-shaped GES in the WT and S272E phosphomimetic transgenic rods appeared well organized, and the rhodopsin content looked vesicular, possibly indicative of a nascent RTC organization. The morphological features of the S272A mutant were quite different; the GESs appeared disorganized, and the rhodopsin content seemed more tubular (Fig. 5D). The mean GES 3D volume in cells expressing the GFP–Rabin8-S272E phosphomimetic was not significantly different from that with GFP–Rabin8-WT [0.4351 µm^3^, *n*=3 versus 0.4688 µm^3^, *n*=4, standard error of difference (s.e.d.)=0.2662, *P*=0.9895] (Fig. 5E). In contrast, the mean 3D GES volume in non-phosphorylatable GFP–Rabin8-S272A-expressing cells (1.369 µm^3^, *n*=3) was significantly different from that for cells expressing GFP–Rabin8-WT (SED=0.2638, *P*=0.0233) and the GFP–Rabin8-S272E mutant (SED=0.2463, *P*=0.0195). This morphological analysis of individual transgenic photoreceptors indicates that the expression of a non-phosphorylatable Rabin8 adversely affects the size and shape of biosynthetic organelles, particularly the GESs, engaged in rhodopsin trafficking.

### GFP–Rabin8 colocalizes with NDR2 kinase and the RTC-R-SNARE-VAMP7 at the Golgi and the GESs

**Fig. 6.**
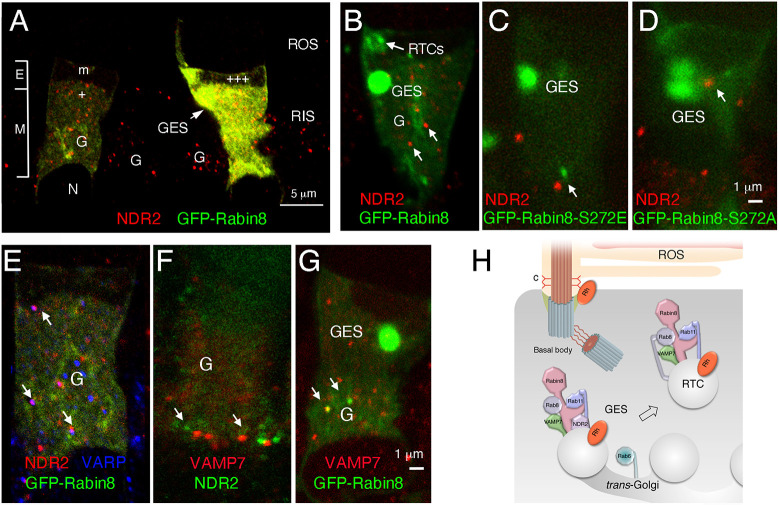
**GFP–Rabin8 colocalizes with NDR2 kinase and RTC-R-SNARE-VAMP7 at the Golgi and the GESs.** (A) Confocal optical section of a transgenic retina expressing GFP–Rabin8 (green) stained with anti-NRD2 (red). NDR2 localized in the Golgi and GES area. (B–D) Transgenic photoreceptors expressing GFP–Rabin8-WT (B), or GFP–Rabin8-S272E (phosphomimetic) (C) or GFP-Rabin8-S272A (non-phosphorylatable) mutants (D) labeled with anti-NDR2 antibody (red). Arrows point to the Golgi (denoted G) and GES locations of NDR2. (E) A transgenic photoreceptor expressing GFP–Rabin8-WT (green) labeled with anti-NDR2 (red) and anti-VARP antibody (blue). (F) A photoreceptor cell labeled with anti-NDR2 (green) and anti-VAMP7 (red). (G) A transgenic photoreceptor expressing GFP–Rabin8-WT (green) labeled with anti-VAMP7 antibody (red). Arrows in E–G point to sites of colocalization or proximity in the Golgi area. Images shown representative of two repeats (H) A rod photoreceptor schematic depicting molecular interactions involved in Rabin8 phosphorylation at the GES and RTC budding, as determined by our study.

As shown in Fig. 3G, NDR2 kinase localized at the tips of the Rab6-positive trans-Golgi cisternae in cells expressing GFP–Rabin8-WT. Notably, NDR2 was detected in the Golgi area both in transgenic rods expressing GFP–Rabin8-WT and, at a comparable level, in the neighboring non-expressing rods (Fig. 6A), indicating that transgene expression did not affect NDR2 expression. Moreover, NDR2 localized similarly at the Golgi and the GESs in transgenic rods expressing GFP–Rabin8-WT and the S272E or S272A mutants (Fig. 6B–D). To characterize further the photoreceptor protein network associated with NDR2 kinase, we examined its colocalization with Rabin8-associated regulators of rhodopsin trafficking. One of these, the VAMP7-binding scaffold protein Vps9-ankyrin repeat protein (VARP; also known as ANKRD27) cooperates with Rabin8 in the ciliary trafficking of the RTC-associated R-SNARE VAMP7 via a tubular network in the Golgi area that might participate in the retrieval of VAMP7 from the RIS plasma membrane following RTC fusion (Kandachar et al., 2018). This VARP function is consistent with its known role in stabilizing VAMP7 in the closed inactive conformation to prevent premature SNARE complex formation in VAMP7 targeting, neurite outgrowth, and retromer-mediated endosomal recycling pathway (Burgo et al., 2009; Fukuda, 2016; Schafer et al., 2012). We found that VARP colocalized or associated with NDR2 (Fig. 6E), suggesting their engagement in the VAMP7-retrieval pathway in the Golgi area, where VAMP7 was also colocalized with NDR2 (Fig. 6F) and GFP–Rabin8-WT (Fig. 6G). Based on this study, we propose that Rab11 recruits Rabin8 to the Golgi or GES membrane where RTC formation is finalized by VAMP7 incorporation in an event that requires NDR2 phosphorylated Rabin8, which controls the RTC release from the Golgi or GESs and subsequent Rab8 activation, as schematically presented (Fig. 6H).

## DISCUSSION

Our study shows that GFP–Rabin8-S272E, mimicking Rabin8 NDR2 kinase-phosphorylated at the GESs, directs Rab8 activation on RTCs in *X. laevis* rods. This crucial event occurs within the Rab11–Rabin8–Rab8 ciliogenesis complex that regulates rhodopsin delivery for the biogenesis of cilia-derived light-sensing organelle, the ROS. It implicates the GESs as checkpoints where NDR2 kinase-phosphorylated Rabin8 switches binding preferences, allowing conventional rhodopsin-bearing membrane progression to the cilium via RTCs. Disruption of these processes likely induces reported rhodopsin mislocalization (Leger et al., 2018). This mechanism might contribute to retinal degeneration and early-onset blindness caused by disruption of NDR2, the product of the canine early retinal degeneration (*erd*) gene.

In this study, we show that both GFP–Rabin8 and NDR2 localize to the tips of the *trans*-Golgi cisternae, and at the *trans*-Golgi-apposed sites that we name Golgi exit sites (GESs). Based on their location and function, GESs likely correspond to the *trans*-Golgi network (TGN), a major secretory pathway sorting station that directs newly synthesized proteins to different subcellular destinations (Griffiths and Simons, 1986). The TGN is a distinct and permanent structural compartment of the Golgi containing one of the few known resident integral membrane proteins TGN38 (also known as TGN41 or TGOLN2) (Luzio et al., 1990). However, although TGN38 is highly conserved in mammals, the species reactivity of available antibodies precludes localization of this established marker in the frog retina. Thus, currently, the identification of the TGN in the frog photoreceptor can only be based on its location and morphology. The seminal 3D structure of the mammalian *trans*-Golgi and the TGN obtained by high-voltage electron microscopy and computer axial tomography has shown that the *trans*-Golgi cisternae are continuous at their ends with tubules contributing to the TGN (Ladinsky et al., 1994). Most vesicular profiles visualized in the TGN with this imaging were connected to TGN tubules and the budding of vesicles appeared to occur synchronously along the length of a TGN tubule. Our previous studies have placed the rhodopsin–Arf4–GBF1 complex, the ASAP1–Rab11–FIP3–Rabin8 complex, and the associated R-SNARE VAMP7 at the *trans*-Golgi-apposed membrane structures that closely fit the morphological description of the TGN (Kandachar et al., 2018; Mazelova et al., 2009a; Wang et al., 2017, 2012). We also previously reported that the morphology of these membrane structures changed upon phospholipid alteration or the transgenic expression of the GFP–VAMP7-R150E mutant in rods, which caused the buildup of vesicular Rabin8-containing membrane clusters and disrupted cellular architecture, as ascertained by CLEM analysis (Deretic et al., 2004; Kandachar et al., 2018; Mazelova et al., 2009a). We now show that Rabin8 and NDR2 colocalize to the tips of the *trans*-Golgi cisternae, at the presumed site of TGN tubule formation. We find some GES or TGN enlargement upon GFP–Rabin8-WT, and phosphomimetic GFP–Rabin8-S272E expression, presumably due to the slowdown in NDR2 Rabin8 processing. However, a significant enlargement and morphological disturbance of the GESs is caused by non-phosphorylatable GFP–Rabin8-S272A, which was not due to its higher expression levels. The similarity of this phenotype to the GFP–VAMP7-R150E mutant phenotype, along with VAMP7 colocalization with NDR2 and GFP–Rabin8-WT shown in Fig. 6, supports the notion that phosphorylated Rabin8 is linked to VAMP7 R-SNARE incorporation into RTCs. In cells expressing GFP–Rabin8 and GFP–Rabin8-S272E, the GES appears to contain a string of uniform rhodopsin-positive globules, possibly embodying nascent RTCs, suggesting that RTC budding occurs *en bloc* along the length of a tubule. By contrast, in GFP–Rabin8-S272A-expressing cells, the expanded GES seems tubular and, consequently, more likely to fragment than form RTC precursors. We postulate that the GES is the TGN of retinal rods, which serves as a checkpoint where rhodopsin sorting into tubules is linked to the recruitment of VAMP7 for subsequent RTC formation and the NDR2 kinase-regulated Rab11–Rabin8–Rab8 ciliogenesis cascade.

The phenotype of Rab8-GEF-deficient Rabin8-F201A mutant that caused the substantial enlargement of RTCs in *Xenopus* rods resembles that of Rab8-T22N GTP-binding-deficient mutant rods, which featured massive accumulation of irregular fusion-incompetent RTCs at the ciliary base triggering rod cell death and retinal degeneration (Moritz et al., 2001). This similarity indicates that activation of Rab8 by Rabin8 controls RTC formation and fusion competence. Rab8 has also been implicated in rhodopsin trafficking in other animal models (Bachmann-Gagescu et al., 2011; Naharros et al., 2018; Omori et al., 2008). The involvement of Rab11, which is crucial for the membrane association of Rabin8 (this study) and RTC budding (Mazelova et al., 2009a), was also confirmed in rhodopsin trafficking in mice, *Drosophila* and polarized epithelial cells (Reish et al., 2014; Satoh et al., 2005; Thuenauer et al., 2014). Additionally, expression of Arf4 mutant deficient in ASAP1-mediated GTP hydrolysis disrupted rhodopsin trafficking and caused retinal degeneration (Mazelova et al., 2009a). Despite these and numerous other publications employing a wide spectrum of methodologies, animal models and cell lines that are in general agreement on the ciliary pathways taken by sensory receptors in ciliated cells, and by rhodopsin in retinal rod photoreceptors, two publications dispute the role of Arf4, Rab8 and Rab11 in these processes in photoreceptors of mouse knockout models (Pearring et al., 2017; Ying et al., 2016). Because mouse Arf4 is a natural mutant in the conserved α3 helix essential for its interaction with rhodopsin, mouse rods might consequently diverge from the highly conserved Arf4-mediated pathway that includes Rab11 and Rab8 (Deretic et al., 2019). The frog model allows precise dissection of stages and molecular machinery involved in rhodopsin trafficking using dominant-negative mutants. In mammalian rods, other trafficking pathways or molecules can compensate for individual components such as Rab11, Rab8 and Arf4. However, that redundancy might be inadequate for the extremely high volume of trafficking in *Xenopus* rods, which exceeds by an order of magnitude that of the rodent rods (Besharse, 1986). Even so, the *Ndr2* mouse knockout causes aberrant rhodopsin localization (Leger et al., 2018). Rhodopsin trafficking through the RIS plasma membrane has been reported in mouse rod photoreceptors (Haggerty et al., 2024), whereas frog rods target RTCs to the ciliary base. The RIS plasma membrane SNARE syntaxin 3 is an essential regulator of rhodopsin trafficking in frogs and mice (Kakakhel et al., 2020; Mazelova et al., 2009b). Its accumulation in ROS, along with other non-ciliary proteins, underlies photoreceptor degeneration in Bardet–Biedl syndrome (Datta et al., 2015). When analyzing processes related to human eye disease, additional suitable animal models could shed light on the similarities and disparities arising from the comparison between the frog and mouse models.

A naturally occurring model for the human retinal degenerative disease Leber congenital amaurosis (LCA), caused by the SINE insertion in exon 4 of the canine *erd* gene encoding NDR2 is predicted to remove a part of the translated protein affecting multiple NDR2 functions, including the kinase activity (Goldstein et al., 2010). This mutation causes photoreceptor cell death followed by the proliferation and formation of hybrid photoreceptors that express both rhodopsin and cone S-opsin that renew their outer segments diffusely like cones (Berta et al., 2011). The mouse *Ndr2* knockout model, in addition to causing rhodopsin mislocalization, also has increased cell proliferation within the inner nuclear layer (Leger et al., 2018). We have observed NDR2 kinase in the deeper retinal layers, where it might play a role in regulating cell proliferation. Future studies should reveal whether endogenous Rabin8 is phosphorylated by NDR2 in frog retinal photoreceptors and whether expressing kinase-deficient mutant NDR2 in transgenic retinas causes Rabin8 accumulation at the GESs like the non-phosphorylatable Rabin8. Our present study focused on the effect of Rabin8 mutants on rhodopsin trafficking and defined, at the molecular level, the Rab11-dual effector pathway and Rabin8 in particular as a main target of the RIS NDR2 kinase, the malfunction of which can undoubtedly contribute to the outcome of (*erd*) gene mutation leading to retinal degeneration and early-onset blindness.

## MATERIALS AND METHODS

### Materials

Myc–Rabin8 and rabbit polyclonal anti-Rabin8 (Hattula et al., 2002) were gifts from Johan Peranen, University of Helsinki, Finland. Antibodies used in this study were as follows: mouse monoclonal antibodies (mAb) anti-Rab11 (610656, BD Biosciences), anti-rhodopsin (11D5) (Deretic and Papermaster, 1991), anti-VAMP7 (MAB6117, R&D Systems), and anti-NDR1/2 (Antibody (E-2): sc-271703, Santa Cruz Biotechnology); rabbit polyclonal antibodies anti-Rab8 (R5530, Sigma), anti-Rabin8 (Hattula et al., 2002) and anti-VARP (24034-1-AP, Proteintech).

### Plasmid construction and mutagenesis

6His-tagged human Rabin8 was constructed by subcloning a *BamHI*-fragment obtained from myc-Rabin8 (a gift from Johan Peranen) into pET-28a vector (EMD Millipore). The same fragment was subcloned into pGEX-KG vector to express GST–Rabin8. The point mutations were introduced using one or two rounds of PCR by site-directed mutagenesis (QuikChange II Mutagenesis Kit, Agilent Technologies). The GST–Rabin8-F201A/S272A mutant and GST-Rabin8-T419A/Y423A/L428A triple mutant were obtained from GenScript Biotech. To construct the Rabin8 expression plasmids for transgenesis, the human Rabin8 cDNA was amplified from GST–Rabin8, derived from Myc–Rabin8 (Kandachar et al., 2018). GFP was amplified from a separate source. After PCR amplification, the fragments were ligated into the EcoRI/NotI sites of the standard XOP0.8 2DI attb eGFP-N1 vector (Tam and Moritz, 2006). A clone containing GFP–Rabin8 was verified by DNA sequencing, linearized with FseI and used for transgenesis. The point mutations were introduced using one or two rounds of PCR by site-directed mutagenesis (QuikChange II Mutagenesis Kit, Agilent Technologies). GFP–Rabin8-F201A/S272A mutant and GFP–Rabin8-T419A/Y423A/L428A triple mutant were obtained from GenScript Biotech.

### Generation of transgenic *X. laevis* expressing Rabin8–GFP fusion proteins

Human Rabin8 was expressed as a GFP fusion protein in transgenic *X. laevis* retinal rod photoreceptors, using a vector containing 0.8 kb of the *X. laevis* rhodopsin promoter, as described (Kandachar et al., 2018; Mazelova et al., 2009a; Tam et al., 2013, 2006). Transgenic *X. laevis* tadpoles were generated by the methods previously described (Kroll and Amaya, 1996; Tam and Moritz, 2006, 2007). Briefly, linearized plasmids containing a Rabin8 expression cassette driven by the *Xenopus* rhodopsin promoter were incubated with sperm before being injected into freshly laid eggs. The resulting embryos were housed in 4-l tanks in an 18°C incubator on a 12-h-light and 12-h-dark cycle. The average light intensity inside the incubator was 1700 lux. At 5 days post-fertilization (dpf), embryos were screened for GFP expression under an epifluorescence dissecting microscope. At 14 dpf (corresponding to developmental stage 48), normally developed transgenic *X. laevis* were euthanized and their eyes were enucleated and fixed in 4% paraformaldehyde buffered with 0.1 M sodium phosphate pH 7.4. Each transgenesis experiment generated ∼10–20 transgenic tadpoles expressing each Rabin8–GFP fusion protein. All animal experiments were performed according to approved guidelines.

### Preparation of photoreceptor-enriched post-nuclear supernatant

*Rana berlandieri* frogs were dark-adapted for 2 h before the experiment. Post-nuclear supernatant (PNS) enriched in photoreceptor biosynthetic membranes isolated from dark-adapted retinas was prepared as described (Deretic and Papermaster, 1991). PNS was frozen and stored in small aliquots.

### Preparation of recombinant proteins

Recombinant proteins were expressed in *E*. *coli* BL21-CodonPlus (DE3)-RIPL (Agilent Technologies) by inducing with 0.1 mM isopropyl β-D-1-thiogalactopyranoside (IPTG, Sigma-15502). Briefly, bacteria transformed with plasmids for protein expression were grown in 250 ml of LB with appropriate antibiotic to an optical density of 600 nm (OD600) of 0.5 and induced with 0.1 M IPTG overnight at room temperature (RT). The overnight culture was centrifuged, and the pellet was resuspended in 15 ml of lysis buffer (2 mM EDTA, 0.1 mg/ml lysozyme, 10 µg/ml DNaseI, and protease inhibitor cocktail in PBS). The bacterial cells were lysed by sonication with 15-s pulses with a 45-s rest between pulses repeated 30 times. The lysate was centrifuged at 18,500 ***g*** for 30 min to remove cell debris. To purify GST-tagged proteins, the cleared lysate was incubated with glutathione–Sepharose 4B beads (GE Healthcare) for 2 h or overnight at 4°C. The beads were washed with PBS and the GST-tagged proteins were eluted with 15 mM reduced glutathione in 50 mM Tris-HCl pH 8.0.

### GST-fusion protein pull-down assay

To analyze direct protein interactions, 6His–Rab11a and 6His–Rab8a (5 µg each), were preincubated with 100 µM GDPβS or GTPγS (Sigma) in 100 µl of nucleotide loading buffer (25 mM HEPES pH 7.4, 100 mM NaCl, 0.5 mM MgCl_2_, 1 mM EDTA, 1 mM ATP and 1 mM DTT) at 30°C for 1 h. Concurrently, 30 μl of glutathione–Sepharose 4B beads were bound to 5 μg of GST or GST fusion proteins, by incubating for 1 h on a rotator at 4°C. Immobilized GST fusion proteins were incubated at RT for 2 h in 500 µl reaction buffer (50 mM HEPES pH 7.4, 150 mM NaCl, 5 mM MgCl_2_·6H_2_O, 0.1% Triton X-100, 0.1% BSA and 1 mM PMSF) with 2–3 μg of purified proteins, as indicated. Glutathione–Sepharose 4B beads were then washed six times with the reaction buffer. Bound proteins were eluted in 20 μl of SDS-PAGE sample buffer and analyzed by SDS-PAGE and immunoblotting.

### Immunoblotting

Proteins were separated by SDS-PAGE on 4–15% TGX gels (Bio-Rad). Gels were blotted onto Immobilon-P PVDF membranes (Bio-Rad) and probed with specific antibodies, as indicated. The blots were blocked with 5% non-fat dry milk (Bio-Rad) in Tris-buffered saline with 0.1% Tween-20 (TBS-T) and probed with anti-Rab8 (1:500), anti-Rab11 (1:500), anti-GST (1:2000) or anti-NDR2 (1:200) antibodies. The blots were incubated with secondary antibodies conjugated to HRP (Thermo Fisher Scientific). Bound antibodies were detected using SuperSignal West Pico PLUS Chemiluminescent Substrate (Thermo Fisher Scientific), visualized using ChemiDoc Imaging System (Bio-Rad), and quantified using Image Lab 6.1 (Bio-Rad).

### Confocal microscopy

Confocal microscopy was performed on *Xenopus laevis* retinas as previously described (Mazelova et al., 2009a). 100 μm vibratome retinal sections were blocked for 1 h at RT in antibody buffer (1% BSA, 0.3% Triton-X in PBS) and labeled with the following primary antibodies: rabbit polyclonal: anti-Rabin8 (1:100); anti-Rab6 (1:100); anti-VARP (1:100); mouse monoclonal: anti-rhodopsin (11D5) (1:200), anti-VAMP7 (1:100) or anti-NDR2 (1:100), and GFP-nanobody-Alexa647 conjugate NbGFP-A647 (Haggerty et al., 2024). The secondary antibodies (1:200) were goat anti-rabbit-IgG or goat anti-mouse-IgG conjugated to Cy3 (Jackson ImmunoResearch Laboratories) or Alexa Fluor 488 and 647 (Thermo Fisher Scientific). Nuclei were counterstained for 10 min at RT with TO-PRO-3 (1:500; Molecular Probes) and sections were mounted on slides with Vectashield (Vector Laboratories). Confocal optical sections were generated on a Zeiss LSM 800 AiryScan Confocal Microscope (Carl Zeiss, Inc), mounted on Inverted (AxioObserver) microscope, using Plan-Apochromat 40*×*/1.4 Oil *DIC* M27 (Carl Zeiss, Inc). For WGA labeling, samples were prepared for confocal microscopy as previously described (Tam et al., 2013). Briefly, contralateral eyes were fixed in 4% paraformaldehyde buffered with 0.1 M sodium phosphate pH 7.4. Fixed eyes were infiltrated with 20% sucrose for 2–6 h, embedded in OCT (Sakura Finetek), frozen and stored at −80°C before sectioning. 12 µm slices were obtained using a cryostat and labeled overnight with Alexa Fluor 555-conjugated wheat germ agglutinin (WGA; Thermo Fisher Scientific) and Hoechst 33342 (Sigma-Aldrich) counterstain, and mounted with Mowiol medium (Sigma Aldrich). Labeled cryosections were imaged using a Zeiss LSM 880 Airyscan microscope with a 40× NA 1.4 water immersion objective (Carl Zeiss, Oberkochen, Baden-Württemberg, Germany). All digital images were prepared, and overall brightness and contrast were adjusted, using Adobe Photoshop CS4 (Adobe Systems Inc.). For quantitative analysis, a grid of lines was placed on the confocal optical sections over the ellipsoid or myoid areas of transgenic RISs, and the surface density of GFP–Rabin8-positive membranes (Sv^GFP-Rabin8^) was calculated from the number of their intersections with the index lines of known length (Weibel and Bolender, 1973).

### Structured illumination microscopy

Either 8 μm cryosections or 100 μm vibratome sections of GFP–Rabin8 transgenic *Xenopus laevis* tadpole eyes were prepared for SIM. Sections were first labeled with either WGA-Cy3, anti-rhodopsin 11D5 antibody (Deretic and Papermaster, 1991) or the GFP-nanobody–Alexa Fluor 647 conjugate NbGFP–A647 (Haggerty et al., 2024), then ethanol dehydrated and embedded with Ultrabed resin (Electron Microscopy Sciences, EMS cat# 14310). 1-μm-thick resin sections of the labeled vibratome sections were cut on a Leica UCT ultramicrotome and dried onto #1.5 glass coverslips. All SIM samples were mounted in ProLong Glass (Thermo Fisher Scientific, cat# P36980). SIM imaging was performed at room temperature using a Nikon-N-SIM-E microscope system equipped with a Hamamatsu Flash 4.0 camera and an SR-HP Apochromat TIRF 100*×*/NA 1.49 oil objective. *Z*-projections were collected across the section (containing 15 to 20 slices), using 0.2 μM *z*-section thickness. Samples were imaged using 488 nm and 647 nm laser settings with 15 grating pattern images. Hoechst 33342 nuclear staining was captured using the widefield 405 nm camera setting and overlayed onto sample images during analysis. SIM reconstructions and 3D-deconvolution of SIM reconstructed z-stacks were performed using Nikon NIS-Elements software.

### Image analysis

SIM *z*-stack projections were processed using Fiji/ImageJ software (Schindelin et al., 2012). For visualization, *z*-stacks were processed as maximum intensity projections, and brightness and contrast were adjusted for image clarity. Volumes from individual GES puncta in SIM *z*-stacks were calculated using the 3D suite plugin in Fiji/ImageJ after half-maximum intensity thresholding.

### Statistical analysis

GraphPad Prism 10 for macOS Version 10.2.2 was used to perform statistical analysis and generate plots.
